# Staufen1-mediated mRNA decay induces Requiem mRNA decay through binding of Staufen1 to the Requiem 3′UTR

**DOI:** 10.1093/nar/gku388

**Published:** 2014-05-05

**Authors:** Min Young Kim, Jungyun Park, Jong Joo Lee, Dae Hyun Ha, Jonghwan Kim, Chan Gil Kim, Jungwook Hwang, Chul Geun Kim

**Affiliations:** 1 Department of Life Science and Research Institute for Natural Sciences, College of Natural Sciences; 2 Graduate School for Biomedical Science and Engineering, Hanyang University, Seoul 133-791, Korea; 3 Department of Biotechnology, Konkuk University, Chungju 380-701, Korea

## Abstract

Requiem (REQ/DPF2) was originally identified as an apoptosis-inducing protein in mouse myeloid cells and belongs to the novel Krüppel-type zinc finger d4-protein family of proteins, which includes neuro-d4 (DPF1) and cer-d4 (DPF3). Interestingly, when a portion of the REQ messenger ribonucleic acid (mRNA) 3′ untranslated region (3′UTR), referred to as G8, was overexpressed in K562 cells, β-globin expression was induced, suggesting that the 3′UTR of REQ mRNA plays a physiological role. Here, we present evidence that the REQ mRNA 3′UTR, along with its *trans*-acting factor, Staufen1 (STAU1), is able to reduce the level of REQ mRNA via STAU1-mediated mRNA decay (SMD). By screening a complementary deoxyribonucleic acid (cDNA) expression library with an RNA–ligand binding assay, we identified STAU1 as an interactor of the REQ mRNA 3′UTR. Specifically, we provide evidence that STAU1 binds to putative 30-nucleotide stem–loop-structured RNA sequences within the G8 region, which we term the protein binding site core; this binding triggers the degradation of REQ mRNA and thus regulates translation. Furthermore, we demonstrate that siRNA-mediated silencing of either STAU1 or UPF1 increases the abundance of cellular REQ mRNA and, consequently, the REQ protein, indicating that REQ mRNA is a target of SMD.

## INTRODUCTION

Requiem (REQ), also known as DPF2, ubi-d4 or BAF45D, was initially identified as an essential gene for apoptosis following IL-3 deprivation in myeloid cells and was named for its role in cell death (1). REQ is a member of the d4-protein family of proteins, which also includes neuro-d4 (DPF1, BAF45B), cer-d4 (DPF3, BAF45C) and a more distantly related protein, PHF10 (BAF45A) (2,3). Neuro-d4 and cer-d4 are known to be expressed in the central and peripheral nervous systems of vertebrates, whereas REQ is equally expressed in all other tissues and organs (1–2,4). All proteins in this family consist of a d4-protein family-specific N-terminal 2/3 domain, a Cys2His2 (C2H2) zinc finger (ZF) or a Krüppel-type ZF in the central part of the protein and a C-terminal tandem plant homeodomain (PHD) (5–7). REQ was recently reported to link RelB/p52 and the Brn-type Switch/Sucrose NonFermentable (SWI/SNF) complex with NF-κB transactivation via its noncanonical pathway (8), whereas all other d4-protein family members function as efficient coactivators of the RelA/p50 dimer at the most downstream part of the NF-κB canonical pathway (9). In previous studies, we intriguingly identified REQ as a putative globin switching factor (10,11). In addition, expression of human adult-type β-globin has been shown to be induced in transient heterokaryons of human K562 and mouse erythroleukemia (MEL) cells (12,13). Since K562 cells express fetal-type γ-globin but not adult-type β-globin before heterokaryon formation with MEL cells, the expression of human β-globin in K562 cells has been speculated to be induced by some factor(s) present in MEL cells. Based on these speculations, we screened for K562 cell phenotypes induced by the expression of MEL cell-derived complementary deoxyribonucleic acid (cDNA). We isolated a clone we termed G8, which encodes a portion of the 3′-untranslated region (3′UTR) of REQ, as a putative factor involved in γ- to β-globin switching.

Since the overexpression of G8 appeared to mediate globin switching, we speculated that 3′UTR-mediated translational control could also play a role in the regulation of REQ gene expression. Indeed, when REQ was initially identified as a proapoptotic factor by screening for phenotypes induced by antisense cDNA expression (1), a partial cDNA encoding the 3′UTR of REQ was found to protect against apoptosis by the antisense suppression of an endogenous 2.4-kb REQ messenger ribonucleic acid (mRNA). Thus, it is conceivable that the regulation of REQ gene expression is modulated by a 3′UTR-mediated translational control mechanism.

The contributions of 3′UTRs to translation regulation can involve a number of different mechanisms, including poly(A) length, the presence of a cytoplasmic polyadenylation element (CPE), specific RNA binding elements and microRNAs (miRNAs) [see (14) for review]. The recognition and binding of proteins or protein complexes to defined sequences or structural RNA elements in the mRNA 3′UTR make up a common translational control mechanism [see (15) for review]. This mechanism is generally controlled by stimulus-dependent post-translational modifications that regulate protein–protein interactions and complex assemblies, as well as the RNA-binding properties of these assemblages, which cumulatively regulate gene expression either by altering mRNA stability or by influencing the translational efficiency. As a post-transcriptional regulator, Staufen (STAU) has been implicated in mRNA localization during *Drosophila* embryogenesis and in mammalian neurons (16–18). In addition, STAU, a double-stranded RNA-binding protein, is also known to recognize the Staufen-binding site (SBS) in many 3′UTRs and thereby mediate mRNA decay, a process known as Staufen1-mediated mRNA decay (SMD) (19). The SBS is formed either by intramolecular base pairing in the 3′UTR or by intermolecular base pairing through the interaction of the target mRNA 3′UTR with noncoding RNA (20,21). SMD is a representative post-transcriptional regulation mechanism sharing many similarities with nonsense-mediated mRNA decay (NMD), including a common factor, upframeshift factor 1 (UPF1) [(22) for review]. Briefly, STAU and UPF1 bind to the SBS, at which UPF1 helicase activity but not ATPase activity is necessary (23). Mammalian STAU has two paralogs, STAU1 and STAU2, both of which bind UPF1 with different binding activities and form homodimers and heterodimers with each other before ultimately mediating SMD (23). Recently, SMD was shown to downregulate an anti-adipogenic factor, Krüppel-like factor 2, during adipogenesis, thereby inducing efficient differentiation (24). Intriguingly, STAU1 also appears to function in the mRNA export of a 3′UTR containing inverted repeat Alu elements (IR*Alus*) (20). STAU1 binds to the 3′UTR IR*Alus* region by preventing the binding of protein kinase R to its substrate and thus inhibiting nuclear retention, which ultimately promotes the nuclear export of mRNA (20).

In this study, we show that specific RNA sequences residing in the 3′UTR of REQ, which we cumulatively term the protein binding site (PBS) core, trigger REQ mRNA decay. We present RNA electrophoretic mobility shift assay (EMSA) results demonstrating that, when sequences in the PBS core are individually mutated or deleted, the ability of this region to form complexes with proteins is greatly reduced. We identify STAU1 as one of the factors that binds to the PBS core in the G8 region. Furthermore, we provide evidence that STAU1, which mediates SMD, triggers the degradation of REQ mRNA and regulates translation. Thus, our data indicate that REQ mRNA is an SMD target and also suggest that the PBS core in the G8 region of the REQ 3′UTR is a key regulatory element in SMD.

## MATERIALS AND METHODS

Detailed information regarding plasmid construction, induction tests of the Tet-on regulator, flow cytometry, *in vitro* transcription, cell fractionation, ultraviolet (UV)-induced crosslinking of RNA, recombinant expression and purification of hSTAU1-GST and RNA ligand-based cDNA expression library screening is provided in the Supplementary Materials and Methods section.

### Cell culture

The human chronic myelogenous leukemia cell line K562, the human cervical adenocarcinoma cell line HeLa, the MEL cell line DS19 and derivatives of these lines harboring various reporter constructs were maintained in a humidified atmosphere of 5% CO_2_ at 37°C. K562 cells were cultured in RPMI 1640 (GIBCO/BRL), whereas HeLa and MEL lines were cultured in Dulbecco's modified Eagle's medium; all medium was supplemented with 10% bovine serum (Hyclone) and 1% penicillin/streptomycin. Terminal differentiation of MEL cells was achieved by supplementing the medium with the chemical inducer hexamethylene-bis-acetamide (5 mM).

### Cell transfection and generation of the K562 172-1 stable cell line

HeLa cells were transfected with plasmid DNA or siRNA using Lipofectamine 2000 (Invitrogen) or Oligofectamine (Invitrogen), respectively. One day after siRNA transfection, cells were retransfected with various pFLuc variants [pGL3-G8, -3′UTR, -ΔPBS, or –poly(A)] harboring the firefly luciferase gene or with pRLuc containing the *Renilla* luciferase gene (as a control). When specified, HeLa cells were incubated with a transcriptional inhibitor, 5, 6-dichloro-1-β-D-ribofuranosylbenzimidazole (DRB, 100 μg/ml), for various time points 3 days after siRNA transfection. For siRNA-mediated silencing of UPF1, UPF2 and STAU1, siRNA sequences were employed as previously described (24,25). Commercially available control siRNA was also used (Bioneer).

To generate stable K562 cell lines expressing the Tet-on regulator, 10 μg of the linearized Tet-on regulator plasmid, p172-1 (a kind gift from Dr Manfred Gössen), was electroporated into K562 cells at conditions of 50 μF, 500 volts x 2. Positive cells were selected in the presence of G418 (200 μg/ml). After 2 weeks of sib-selection in 96-well plates, selected clones were assessed for the presence of p172-1 by monitoring the reporter activity after transient transfection of either pTRE-Luc (Clontech) or pTRE-EGFP (enhanced green fluorescence protein) into the cells.

### RNA preparation and semi-quantitative or quantitative reverse transcriptase-polymerase chain reaction (RT-sqPCR or RT-qPCR)

Total cellular RNA was prepared using Trizol (Invitrogen) according to the manufacturer's protocol. To eliminate possible contamination by exogenous or endogenous DNA, the RNA was treated with RQ DNase I (Promega) for 30 min at 37°C before continuing with the RNA extraction protocol. The quantity and quality of the isolated RNA were confirmed using UV spectroscopy (Shimadzu, Japan) and electrophoresis on a 1% agarose gel, respectively. For RT-PCR, cDNA was synthesized using random hexamers (Invitrogen); reverse-transcribed cDNA was then amplified by the PCR using various sets of exon-specific primers. To amplify cDNA, sqPCR was performed using GoTaq polymerase (Promega). Primers used to PCR-amplify EGFP and hHPRT RNA were 5′-CAC CCT GGT GAA CCG CAT CG-3′ (sense) and 5′-GCT GCA CGC TGC CGT CCT CG-3′ (antisense) and 5′-GCT GGT GAA AAG GAC CCC A-3′ (sense) and 5′-AGC TCT ACT AAG CAG ATG GC-3′ (antisense), respectively. The pFLuc and pRLuc mRNAs were amplified by RT-qPCR using a CFX96 system (Bio-Rad) and Fast SYBR Green Master Mix (Applied Biosystems). cDNA synthesized from HeLa cells transfected with RLuc and FLuc mRNA was amplified using the primer pairs 5′-ATA TTG AGC CAG TAG CGC GG-3′ (sense) and 5′-GCC AAA CAA GCA CCC CAA TC-3′ (antisense) and 5′-AAT GTC CGT TCG GTT GGC-3′ (sense) and 5′-GTA GGC TGC GAA ATG CCC-3′ (antisense), respectively. The primers used to amplify endogenous REQ and GAPDH mRNA were 5′-TTT GTG ATG ACT GCG ATC GG-3′ (sense) and 5′-GGT GAG GGT GAT GTA AGC AG-3′ (antisense) and 5′-CAA GAT CAT CAG CAA TGC C-3′ (sense) and 5′-CTG TGG TCA TGA GTC CTT CC-3′ (antisense). C-Jun and IL-7R mRNA were amplified as previously described (26).

### RNA EMSA

*In vitro* transcribed [α-^32^P]-labeled RNA (100 000–500 000 cpm) was heated at 95°C for 5 min and subsequently allowed to cool to room temperature for 30 min. Protein extracts were incubated with the RNA probe in a buffer containing 30-mM Tris (pH 7.4), 3-mM MgCl_2_, 70-mM KCl and 1.5-mM Dithiothreitol (DTT). Then, 10 μg of protein was incubated with heparin sulfate (5 mg/ml) in a total volume of 15 μl. The reaction was carried out at room temperature for 30 min. To discriminate heterogeneous secondary-structured RNA probes from the specific RNA–protein complexes and to estimate the molecular weights of the RNA–protein complexes using UV-induced crosslinking, RNase T1 and RNase A were added at final concentrations of 3000 U/ml and 10 μg/ml, respectively, and the mixture was incubated for another 15 min at room temperature. Samples were resolved on a 5% polyacrylamide gel containing 5% acrylamide-bisacrylamide (29:1) and 0.05% NP-40. For supershift assays, antibodies specific for STAU1 (Abcam) were added to the reaction.

### Computer prediction of RNA secondary structure

The optimal secondary structures for wild-type and truncated RNAs were predicted using the RNAfold web server (rna.tbi.univie.ac.at/cgi-bin/RNAfold.cgi). Minimum free energy and partition function were set as the fold algorithms and basic options, and interactive RNA secondary structure plot and RNA secondary structure plot with reliability annotation were set as the output options.

### Western blotting

Proteins in cell lysates were resolved by electrophoresis on 6–10% polyacrylamide gels and subsequently transferred to PVDF membranes (Millipore). The following antibodies were used: anti-EGFP (Clontech), anti-β-actin (Santa Cruz), anti-GST (Sigma), anti-UPF1 (Cell Signaling), anti-UPF2 (Santa Cruz Biotechnology), anti-calnexin (Santa Cruz Biotechnology), anti-REQ (in-house manufactured anti-rabbit peptide antibodies raised against the peptide Ac-LGEFPVSNSRARC-NH_2_, Mimotopes) and anti-PLCγ (Santa Cruz Biotechnology). Antibodies were diluted 1:200–1:1500 in 5% non-fat dry milk in 0.1% Tween 20/TBS and then incubated overnight at 4°C with rocking. Immunoreactive bands were then visualized using ECL reagents (Thermo).

### RNA immunoprecipitation

RNA immunoprecipitation (IP) was performed as previously described (27), with some modifications. An RNA probe (100 000–500 000 cpm) was used instead of a DNA probe; other reaction conditions were the same as those described above for the RNA EMSA. Antibodies specific for GST (Sigma) and STAU1 (Abcam) were employed for IP. The labeled RNA probe was eluted from precipitated RNA–protein complexes in 50-mM Tris-HCl, 10-mM ethylenediaminetetraacetic acid and 1% sodium dodecyl sulfate (SDS) for 1 h at 65°C. The radioactivity of the eluted probe was then measured by scintillation counting.

## RESULTS

### Clone G8, which encodes a region of the REQ 3′UTR, regulates REQ mRNA abundance

**Figure 1.**
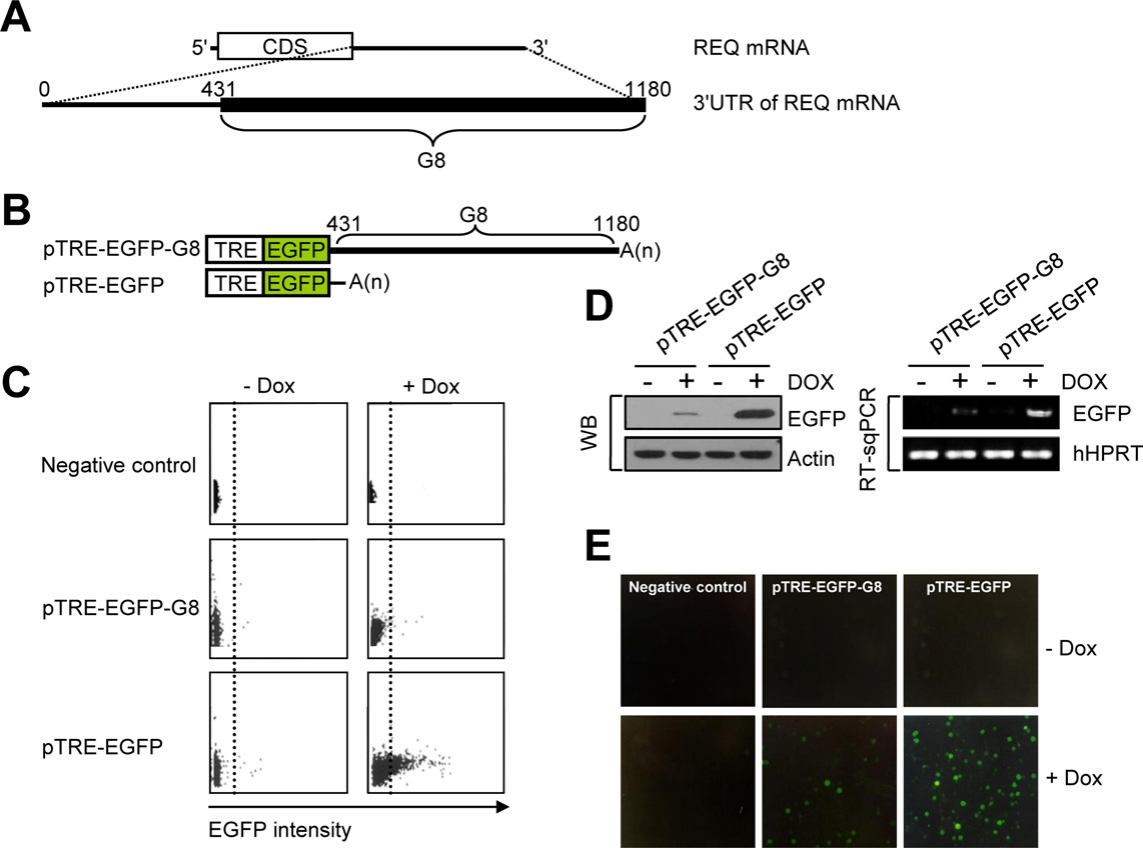
The G8 region in the REQ 3′UTR is involved in mRNA turnover. (**A**) Sequence map of REQ mRNA and the G8 region, which spans from the middle to the end of the 3′UTR. (**B**) Schematic representations of pTRE-EGFP-G8, containing both G8 and EGFP, and the positive control, pTRE-EGFP. Both plasmids contained EGFP. (**C**) Expression of either EGFP-G8 or EGFP in the appropriate K562 Tet-on cell line was induced with 1-mM doxycycline (Dox). After a 48-h induction, cells were harvested and assayed for EGFP activity by fluorescence-activated cell sorting. (**D**) Western blotting and semi-quantitive RT-PCR (RT-sqPCR) were employed to determine the expression of EGFP. Actin (WB) and hHPRT (RT-sqPCR) served as controls for variations in protein and RNA loading, respectively. (**E**) EGFP was visualized by immunofluorescence microscopy in the stable cell lines. Nontransfected cells served as negative controls.

An antisense sequence of the REQ 3′UTR was previously reported to rescue the FDCP-1 cell line from apoptosis (1); moreover, overexpression of G8 (Figure 1A), corresponding to the sense REQ mRNA 3′UTR, was shown to induce expression of β-globin in K562 cells (10,11). We hypothesized that the REQ mRNA 3′UTR exerts an important role in the regulation of REQ expression at the post-transcriptional level. To test this hypothesis, we determined the effect of the REQ 3′UTR on EGFP expression using a Tet-on inducible system. Cell lines constitutively expressing the Tet regulator protein were generated by transfecting parent K562 cells with p172-1 (28,29). Three clones were sib-selected, and the ability of each clone to drive the expression of Tet-driven reporter plasmids, transiently transfected into the clones, was determined. Cells were transfected with the reporter plasmids pTRE-Luc or pTRE-EGFP, grown in doxycycline to induce expression of the reporter proteins, and the fold inductions were determined 48 h after doxycycline-mediated induction. We selected the cell line showing the highest fold induction in relative luciferase units (276-fold), with minimal background; induction was also Tet concentration dependent (data not shown). Cell lines stably harboring two different reporter constructs, one containing the G8 sequence fused to EGFP (pTRE-EGFP-G8) and one lacking the G8 sequence (pTRE-EGFP; Figure 1B), were established by transfecting each construct into the selected Tet regulator-expressing K562 cell line and selecting for stable cell lines. The effect of the G8 sequence on the expression of EGFP was determined by comparing the levels of EGFP in the two cell lines by flow cytometry. After 48 h of doxycycline-mediated induction, cells were harvested and assayed for EGFP activity. Flow cytometry analysis revealed that G8 dramatically reduced the production of EGFP compared with the level of EGFP in the cells lacking G8 (Figure 1C). To confirm the flow cytometry data, western blotting (WB), RT-sqPCR (Figure 1D) and immunofluorescence (Figure 1E) analyses were performed. Western blot analysis showed that the level of EGFP was significantly reduced in lysates made from Tet-induced cells stably expressing both EGFP and G8 (pTRE-EGFP-G8) compared with Tet-induced cells expressing EGFP alone (pTRE-EGFP) (Figure 1D, left panel). Consistent with these results, the level of EGFP mRNA was lower in cells expressing EGFP-G8 compared with cells expressing EGFP alone (Figure 1D, right panel), indicating that the lower level of EGFP protein was due to a lower level of EGFP mRNA. In addition, Tet-induced cells expressing G8 also showed reduced EGFP expression by immunofluorescence analysis (Figure 1E). Cumulatively, all of these data suggest that the G8 region of the REQ 3′UTR functions in mRNA turnover, which consequently leads to reduced protein expression.

### Proteins in K562 cells specifically bind to the PBS within the 3′UTR of REQ RNA

**Figure 2.**
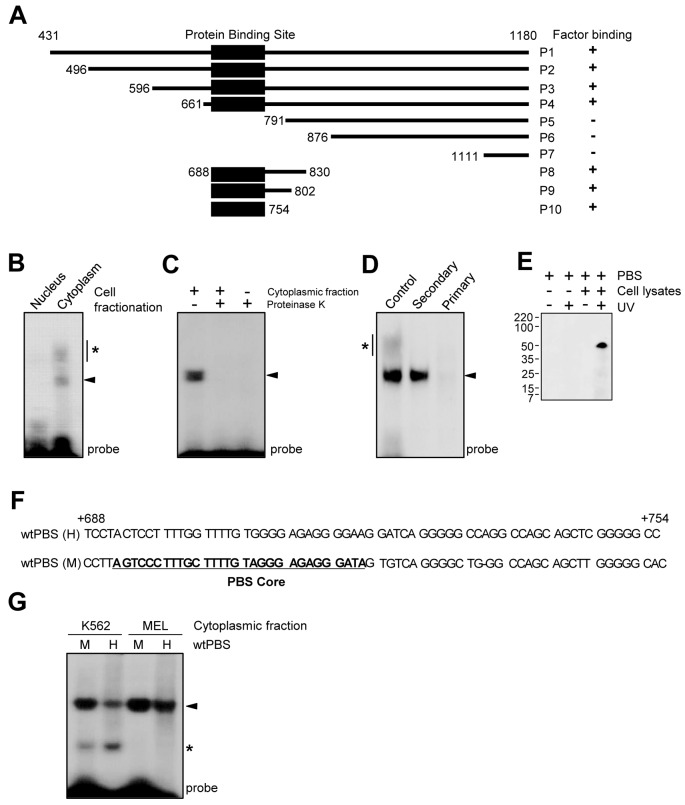
One or more protein(s) in the K562 cell lysate binds to the protein binding site (PBS) in G8. (**A**) Construction of G8 deletion mutants and analysis of RNA–protein interactions within K562 cell extracts. To identify the core protein binding motif, RNA EMSAs were performed with riboprobes corresponding to various truncated versions of G8. The binding activities of these probes are summarized on the right panel. (**B**) RNA EMSAs using either the nuclear or cytoplasmic K562 fraction, along with the radiolabeled PBS riboprobe. (**C**) RNA EMSAs using the K562 cytoplasmic fraction and the radiolabeled PBS riboprobe, performed either in the presence or absence of proteinase K. (**D**) RNA EMSAs performed using the K562 cytoplasmic fraction with either slowly cooled (secondary-structured) or rapidly cooled (primary-structured) radiolabeled PBS riboprobes. The control lane shows the results of an RNA EMSA using PBS riboprobes that did not undergo temperature treatment. (**E**) UV-induced crosslinking of the riboprobe with the cellular protein. The radiolabeled PBS riboprobe was incubated either in the presence or absence of the K562 cytoplasmic fraction. The reaction mixture was then irradiated with UV light for 10 min followed by treatment with a mixture of RNase T1 and RNase A. The crosslinked proteins were resolved by sodium dodecyl sulphate-polyacrylamide gel electrophoresis (SDS-PAGE). (**F**) Highly conserved sequences (the PBS cores) are underlined. H, human; M, mouse. (**G**) RNA EMSAs were performed with either K562 or MEL cell lysates and the radiolabeled PBS riboprobe. Notably, in addition to a specific band (arrow heads), larger or smaller band shift products (asterisks) also appeared in some reactions, suggesting that the RNA–protein complex contains dynamically interacting proteins in addition to a core protein.

mRNA turnover is often mediated by proteins that bind to mRNA, especially the 3′UTR region [reviewed in (14–15,30)]. However, miRNA is also able to downregulate mRNA translation. To exclude this possibility, we performed an *in silico* screen for conserved miRNAs predicted to target the REQ PBS (see below for definition) using various miRNA-matching algorithms (http://www.targetscan.org and http://www.microrna.org). Through these analyses, we could not identify any putative miRNAs that targeted the PBS (data not shown), suggesting that downregulation of translation may not result from miRNA-mediated mRNA turnover. To further study the mechanism of mRNA turnover mediated by the REQ 3′UTR and to determine which region of the 3′UTR is bound to cellular components, we performed RNA EMSAs using radiolabeled RNA probes consisting of a series of G8-deletion mutants. For this analysis, we generated various deletion mutants of the G8 region; each deletion segment was then subcloned into either pGEM3 or pT7Blue, vectors suitable for the *in vitro* synthesis of RNA probes (Figure 2A). To detect RNA–protein interactions, K562 cell lysates were incubated with an excess of the radiolabeled RNA probe in the presence of heparin sulfate as a nonspecific competitor. We found that probes P1, P2, P3 and P4 each formed one distinctive RNA–protein complex with one or more protein(s) in the cytoplasmic extracts (Supplementary Figure S1A). We also note that the RNA–protein interaction properties of each probe were not changed when unprotected RNA was digested with RNase (Supplementary Figure S1B). In contrast, no detectable binding of probes P5, P6 and P7 to cellular proteins was observed (Supplementary Figure S1A and B). The region shared by all probes engaged in protein binding was roughly defined by probe P10, with which all other protein-binding probes overlapped; this region spanned from +688 to +754 in the G8 sequence (Figure 2A). Thus, we defined the P10 region as the PBS. All of our observations suggest that one or more cellular factors that may regulate RNA turnover bind to the PBS in the REQ 3′UTR.

To determine the active site of the RNA-binding *trans*-acting factor, K562 cell lysates were fractionated into nuclear and cytoplasmic extracts, followed by RNA EMSAs with radiolabeled PBS riboprobes. These assays showed that a cytoplasmic *trans*-acting factor binds to the riboprobe (Figure 2B). Next, to identify the nature of the *trans*-acting factor, we performed EMSAs in either the presence or absence of proteinase K (Figure 2C). The presence of proteinase K completely eliminated the radiolabeled riboprobe complex in EMSAs using the cytoplasmic fraction of K562 cell lysates, suggesting that the *trans*-acting factor consists of one or more proteins. The importance of the RNA secondary structure on the formation of the RNA–protein complex was demonstrated by a simple experiment (Figure 2D). First, the RNA probe was heat-denatured at 90°C and then either rapidly cooled to 4°C to maintain the denatured structure or gradually cooled to room temperature to allow the stable formation of secondary RNA structure. The rapidly cooled RNA probe, which did not possess a secondary structure, did not form an RNA–protein complex (Figure 2D). These observations indicate that the PBS is sufficient for forming a specific RNA–protein complex; furthermore, the RNA secondary structure is critical for forming this complex. The molecular mass of the *trans*-acting factor in the K562 cell lysate that bound to the riboprobe PBS was estimated using UV-induced crosslinking. As shown in Figure 2E, one protein of ∼55 kDa was detected in association with the riboprobe that contained the PBS. No bands were observed when the cell lysates or UV was omitted.

Given the high degree of homology between the human and mouse REQ 3′UTR sequences (Figure 2F), we hypothesized that the elements involved in REQ mRNA turnover might be conserved. To determine whether the PBS in the human REQ 3′UTR also formed an RNA–protein complex, we isolated the corresponding human PBS and subcloned it into pGEM3, a vector suitable for the *in vitro* synthesis of RNA probes. As shown in Figure 2G, when each PBS was incubated with an extract from either K562 cells (human) or MEL cells (mouse) as appropriate, the same PBS–protein complex was observed in both groups. This result implies that the turnover mechanism of REQ mRNA is conserved between humans and mice.

### A *trans*-acting factor recognizes an element of predicted secondary structure within the core region of the PBS

**Figure 3.**
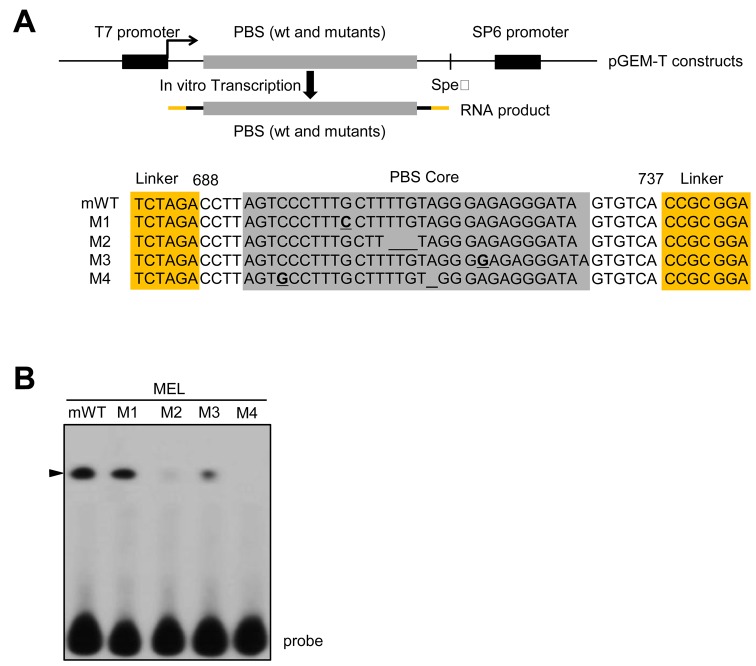
The RNA–protein interaction in the PBS core could be RNA structure dependent. (**A**) Schematic representation of the *in vitro* transcription strategy to obtain RNA products for EMSAs (upper panel). Mutated (M1 and M4), deleted (M2 and M4) and inserted (M3) nucleotides are marked in bold font or underlined. The PBS core is highlighted in gray. Regions highlighted in orange represent sequences from the parent plasmid for *in vitro* transcription. (**B**) Cytoplasmic fractions from MEL cells were incubated with the radiolabeled WT PBS or mutant versions thereof. RNA EMSAs were then performed to investigate RNA–protein interactions. The specific RNA–protein complex is designated with an arrowhead.

**Figure 4.**
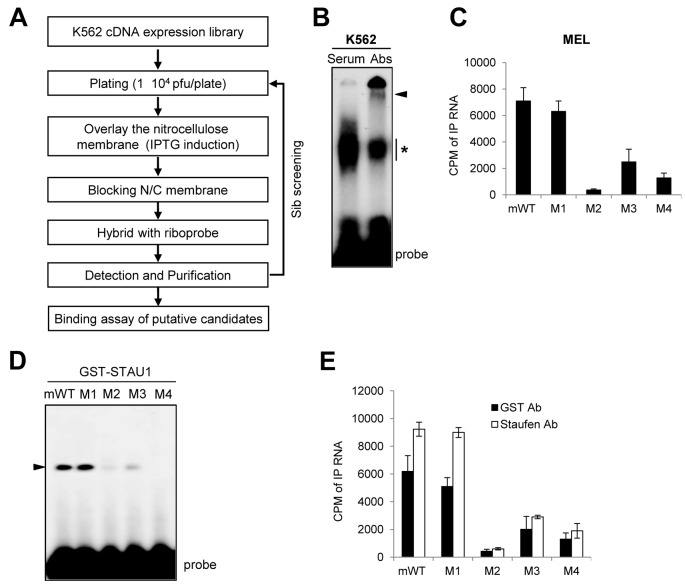
STAU1 is a component of the RNA–protein complex. (**A**) Schematic flow chart of the screening approach used to identify proteins binding to the PBS core in the REQ 3′UTR. (**B**) RNA EMSAs showing that STAU1 binds to the PBS in the REQ 3′UTR. Cytoplasmic extracts from K562 cells were incubated with the radiolabeled PBS probe in either the presence or absence of anti-STAU1 antibodies (Abs). As a negative control, non-immune serum was used. An arrowhead and an asterisk indicate the RNA–STAU1–STAU1 Ab complex and the RNA–STAU1 complex, respectively. (**C**) MEL cell extracts were incubated with various radiolabeled RNA probes, including mWT or mutant versions thereof (M1–M4). Immunoprecipitations were then performed with anti-STAU1 antibodies, and the amounts of co-immunoprecipitated RNA probes were determined by quantifying the radioactivity (CPM) present in the eluates. Columns and error bars represent the mean and standard deviation of at least three independent experiments. (**D**) Recombinant GST-STAU1 was incubated with various radiolabeled RNA probes, including mWT or mutant versions thereof (M1–M4). Protein–RNA complexes were then resolved on a 6% native polyacrylamide gel. An arrowhead indicates the specific RNA–GST–STAU1 complex. (**E**) RNA immunoprecipitation assays using recombinant GST-STAU1 as in (D), except that RNA–protein complexes were immunoprecipitated with either anti-GST or anti-STAU1 antibodies. The amounts of co-immunoprecipitated RNA probes were determined by quantifying the radioactivity present in each of the eluates. Columns and error bars represent the mean and standard deviation of at least three independent experiments.

Our data indicate that the human and mouse PBS both interact with the same cellular protein (Figure 2G). In addition to this finding, the REQ 3′UTR sequences share similarities greater than 64% among 11 mammalian species; moreover, the human and mouse PBS sequences share 71.7% identity, as determined by sequence alignments (Supplementary Figure S2A and B). However, the highly conserved 30-nt RNA sequences within both the human and mouse PBS showed 73.3% identity and 83.3% similarity; thus, we termed this region of the PBS the PBS core (Figure 2F, underlined; Supplementary Figure S2C). Notably, the sequence identities shared by the PBS cores from various mammalian species are greater than 73% (Supplementary Figure S2C). The RNA structure for the mouse PBS core is predicted to contain a hairpin structure composed of an 8-bp central stem, a 1-bp mismatch in the middle, and a terminal stem–loop structure consisting of a 3-bp stem and a 4-nt loop (Figure 3A and Supplementary Figures S2D and S3). This predicted structure prompted us to test whether the PBS core itself is sufficient for binding of the *trans*-acting factor. RNA EMSAs, using the PBS core as the RNA probe, revealed that the PBS core alone was indeed sufficient for RNA–protein complex formation (Figure 3B, first lane; see also Figure 4C–E). Surprisingly, the predicted RNA structure of the human PBS core was not the same as that of the mouse PBS core. The human PBS core was predicted to contain only a stem–loop structure harboring an 8-bp stem and an 11-nt loop, without a mismatching sequence, unlike the mouse PBS core (Supplementary Figure S2D). Since our data indicated that both the human and mouse PBS bind the same cellular factor (Figure 2G), we inferred that the two PBS core sequences should share a conserved RNA structure that mediates this binding. Intriguingly, the RNA structures of the PBS cores of various mammals were all predicted to share a stem–loop harboring longer than a 3-bp stem with a loop of varying sizes (Supplementary Figure S2D), suggesting that the cellular protein is capable of recognizing this RNA structure. To determine whether putative RNA structure of the PBS core is important for protein binding, four versions of the mouse PBS core harboring various deletions, insertions or point mutations were generated (Figure 3A). The four mutants are the following: one nucleotide change (G to C) in M1, the deletion of three nucleotides (TTG) in M2, an insertion of a single nucleotide, G, into the middle stem region in M3 and a C to G point mutation combined with the deletion of a single nucleotide, A, in M4 (Figure 3A). The predicted RNA structures and computationally calculated free folding energy (ΔG) values for the variants are listed in Supplementary Figure S3. Although the predicted RNA structure is often inaccurate, it is assumed that changes of nucleotides are thought to lead the modification of RNA structure. Notably, the linker region derived from the plasmid, presumably, did not modify the predicted RNA structure in any case (Figure 3A and Supplementary Figure S3; highlighted in orange in Figure 3A). To determine the binding affinity of the cellular protein to each of these mutant versions of the PBS core, RNA EMSAs were performed using MEL cytoplasmic extracts and mWT or one of the four PBS core variants (Figure 3B). No binding was observed to the M2 or M4 probes, whereas strong binding was detected to the mWT and M1 probes; weak binding was also detected to the M3 probe. These results suggest that potential changes of RNA structure could mediate the binding of the *trans*-acting protein (see also Figure 4C–E).

### STAU1 binds to the PBS core within the REQ 3′UTR

To identify *trans*-acting factors that bind to the PBS core within the REQ 3′UTR, we employed RNA ligand-based cDNA expression library screening (31). Phagemid-based K562 cDNA expression libraries (5 × 10^5^ clones) were initially screened with an [α-^32^P]-labeled G8-RNA ligand to detect specific RNA–protein interactions (Figure 4A and Supplementary Figure S4A). We identified six positive clones in the initial round of screening and four pure clones after sib-screening. These clones included STAU1 (two clones), ribosomal protein SA (RPSA; laminin receptor 1, LAMR1; one clone) and spermatid perinuclear RNA binding protein (STRBP; 74-kDa double-stranded RNA binding protein, p74; one clone) (Supplementary Figure S4B). Although both STAU1 and STRBP are known to be double-stranded RNA binding proteins (32–34), we focused on STAU1 for further study for the following reasons: (i) STAU1 was independently cloned twice and thus strongly detected by the screen; (ii) STAU1 is known to regulate translation and mRNA decay (19,23,35); and (iii) the RNA–protein complex shown in Figure 2E exhibited a molecular weight slightly greater than 55 kDa. We next confirmed that the plasmid rescued from the original phagemid clone, pBK-CMV-hSTAU1, conferred expression of STAU1 protein when transfected into XL1-Blue cells (Supplementary Figure S4C). To confirm that STAU1 binds to the REQ 3′UTR, RNA EMSAs were performed using K562 cytosolic extracts and the radiolabeled PBS riboprobe (Figure 4B). Although the RNA–protein complex band was observed in both lanes, a shift of this band was detected upon the addition of anti-STAU1 antibodies (Abs) (Figure 4B), consistent with the hypothesis that STAU1 is the *trans*-acting factor. Using radiolabeled RNA probes derived from a series of 5′ deletion mutants of G8 (Figure 2A), we also demonstrated that GST-STAU1 interacts with P1-P4 and P8-P10 but not with P5-P7 in RNA EMSAs (Supplementary Figure S5A). These results confirm that STAU1 is the protein that recognizes the PBS core in G8. To confirm that endogenous STAU1 recognizes the PBS core, radiolabeled mWT PBS core and various variants thereof were incubated with MEL cytosolic extracts; next, PBS core RNA–protein complexes were immunoprecipitated using anti-STAU1 antibodies, and the amounts of immunoprecipitated RNA were determined by quantifying the radioactivity (counts per minute, CPM) in the eluates (Figure 4C). These experiments revealed that higher levels of mWT and M1 were co-immunoprecipitated with STAU1 compared with M2 and M4, and that the amount of co-immunoprecipitated M3 was ∼30% that of mWT (Figure 4C). These observations are consistent with the results shown in Figure 3B and further support the idea that STAU1 is the cellular protein that interacts with the PBS core. Furthermore, we also confirmed that STAU1 binds to the PBS core by RNA EMSA and RNA IP assays. In these assays, we used recombinant GST-tagged STAU1 with either the radiolabeled mWT PBS core or its variants (Figure 4D and E). Consistent with the data shown in Figures 3B and 4C, GST-STAU1 bound to the mWT and M1 probes in RNA EMSAs (Figure 4D). Notably, GST-STAU1 was bound to PBS by UV-crosslinking, consistent with the results in Figure 2E (Supplementary Figure S5B), suggesting that STAU1–RNA complex is crosslinked by UV. Moreover, radiolabeled mWT and M1 were co-immunoprecipitated with GST-STAU1 when either anti-GST or anti-STAU1 antibodies were used in RNA IP assays (Figure 4E), suggesting that GST-STAU1 forms a complex with the PBS core by recognizing its RNA structure. Intriguingly, both mWT and M1 bound strongly to both endogenous STAU1 and GST-STAU1, whereas M3 bound consistently more weakly to endogenous STAU1 and GST-STAU1 in both RNA EMSA and RNA IP assays (Figure 4C–E). On the contrary, the M2 and M4 mutants possessed little or no binding activity compared with mWT and M1. One potential explanation of this finding is that the binding of STAU1 to RNA is dependent on the target RNA structure.

### SMD reduces the level of REQ mRNA in mammalian cells

**Figure 5.**
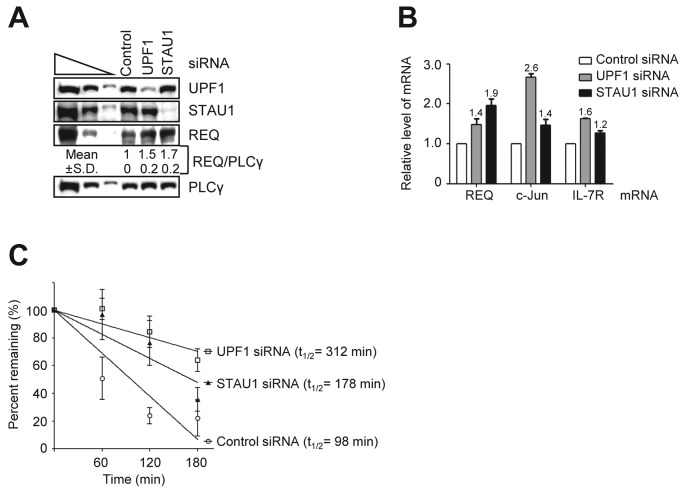
STAU1-mediated mRNA decay (SMD) regulates cellular REQ mRNA decay. (**A**) HeLa cells were transiently transfected with the indicated siRNAs, and cell lysates were then subjected to western blot analysis using the specified antibodies. PLCγ served as an internal control to account for variations in protein loading. The three leftmost lanes show 3-fold dilutions of control siRNA-transfected HeLa cell lysates, demonstrating that the western blot conditions are semi-quantitative. Mean value and standard deviation (SD) were calculated from four independent experiments. (**B**) Total cellular RNA was purified from the cells used in (A), and RT-qPCR was performed. The mRNA levels of REQ, c-Jun and IL-7R (the latter two were used as positive controls for SMD) were normalized to the level of GAPDH mRNA. (**C**) HeLa cells were transiently transfected with control, UPF1 or STAU1 siRNA. After 3 days, DRB was added (100 μg/ml), and cells were incubated for the indicated time periods. Total RNA was then isolated and analyzed by RT-qPCR. Columns (B) or points (C) and error bars represent the mean and standard deviation from at least three independent experiments. The numbers above the bars indicate the mean values from the experiments.

If STAU1 binds to the REQ 3′UTR, it is plausible that endogenous REQ mRNA is degraded by SMD. To test this hypothesis, we employed HeLa cells, which exhibit higher transfection efficiency than either MEL or K562 cells and have been used in many studies of SMD. We transiently transfected HeLa cells with siRNA targeting either STAU1 and UPF1 (both essential factors in mammalian SMD) or a nonspecific control siRNA (Figure 5A and B). Western blot analysis revealed that siRNA-mediated silencing of UPF1 and STAU1 reduced the levels of these proteins to ∼20% and 10%, respectively, of the amounts found in control cells (the levels of UPF1 and STAU1 were also normalized to the level of PLCγ in order to control variations in protein loading) (Figure 5A). Moreover, silencing of UPF1 did not affect the level of STAU1, and silencing of STAU1 did not affect the level of UPF1 (Figure 5A). When UPF1 and STAU1 were individually depleted, the abundance of endogenous REQ was upregulated by ∼1.5- and 1.7-fold, respectively. These results suggest that SMD reduces REQ expression by degradation of REQ mRNA.

To determine whether STAU1 binding to the REQ 3′UTR leads to SMD, the levels of endogenous REQ mRNA in UPF1- and STAU1-knockdown cells were quantified by RT-qPCR. In these experiments, siRNA-mediated knockdown of either UPF1 or STAU1 upregulated endogenous REQ mRNA by ∼1.5- and 2-fold, respectively (Figure 5B). Two known SMD targets, c-Jun and IL7R (26), were used as positive controls whose levels were also upregulated after siRNA-mediated silencing of UPF1 or STAU1 (Figure 5B). Importantly, the half-life of REQ mRNA was also significantly increased by siRNA-mediated silencing of either UPF1 or STAU1 compared with cells transfected with control siRNA (Figure 5C). UPF1 is known to be a common factor in both SMD and NMD; UPF1 has also been shown to bind to UPF2 in an RNA-independent manner. Moreover, downregulation of UPF2, a factor involved in NMD, has been shown to increase the efficiency of SMD by the subsequent increase of UPF1 binding to STAU1 (26). To determine whether the cellular level of UPF2 affects the abundance of REQ mRNA, UPF2-specific siRNA or control siRNA was transiently transfected into HeLa cells. The relative levels of REQ protein and mRNA were then assessed by WB and RT-qPCR, respectively (Supplementary Figure S6A and B). Consistent with a previous report (26), silencing of UPF2 reduced the levels of REQ protein and mRNA by ∼3-fold. Cumulatively, all of these results indicate that REQ mRNA is an SMD substrate in mammalian cells.

**Figure 6.**
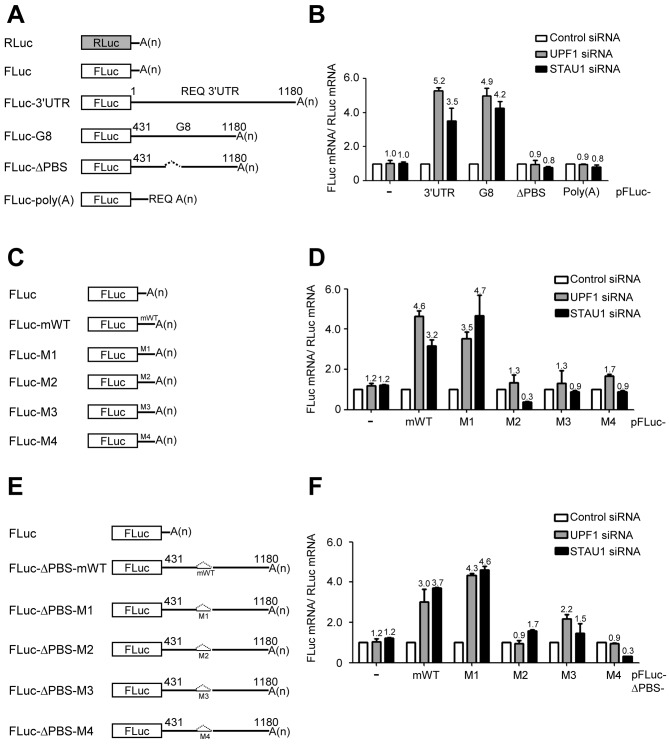
The PBS core in the REQ 3′UTR is required for SMD. (**A**) Schematic representation of constructs harboring various versions of G8 and the firefly luciferase (FLuc) coding sequence; the *Renilla* luciferase (RLuc) plasmid was used as a transfection control. FLuc-ΔPBS and FLuc-poly(A) represent the constructs in which the PBS was deleted and the poly(A) signal of pFLuc was replaced with the REQ poly(A) signal, respectively. (**B**) Comparison of the abilities of various versions of G8 to mediate SMD. HeLa cells were transiently transfected with various FLuc and RLuc reporter plasmids. Total RNA was isolated, and the relative amount of each FLuc mRNA was assessed by RT-qPCR. The level of normalized FLuc mRNA in cells transfected with control siRNA was set to 1. (**C**) Schematic diagrams of FLuc-based constructs containing the mWT PBS core or mutant versions thereof. The mWT PBS core and mutant versions thereof, detailed in Figure 3, were inserted between the stop codon of FLuc and the poly(A) signal. (**D**) As in (B), except that the constructs depicted in (C) were used. (**E**) Schematic diagrams of FLuc-ΔPBS-based constructs containing the mWT PBS core or mutant versions thereof. (**F**) As in (B), except that the constructs depicted in (D) were employed. Columns and error bars represent the mean and standard deviation of at least three independent experiments. The numbers above the bars indicate the mean values from the experiments.

To further investigate the effect of SMD on the REQ 3′UTR, we generated reporter constructs harboring various REQ 3′UTR variants downstream of the firefly luciferase gene, thus replacing the SV40 late poly(A) signal sequences (Figure 6A). HeLa cells were transfected with siRNA against either UPF1 or STAU1 and then transfected with each pFLuc variant and pRLuc as a control. RT-qPCR analysis revealed that siRNA-mediated silencing of STAU1 increased the mRNA levels of pFLuc-3′UTR and pFLuc-G8 by ∼3.5- and 4.2-fold, respectively, compared with cells transfected with control siRNA (Figure 6B). Importantly, silencing of STAU1 did not increase the level of either pFLuc-ΔPBS, which lacked the PBS within G8, or REQ poly(A) mRNA (Figure 6B). As expected, the effect of UPF1 silencing was similar to that of STAU1 silencing (Figure 6A and B). From these data, we conclude that the REQ mRNA is targeted for SMD.

These observations led us to test whether the PBS core itself was necessary for SMD. To this end, we inserted various PBS core variants immediately after the stop codon in pFLuc and then cotransfected each variant with pRLuc into HeLa cells. The levels of FLuc mRNA were then quantified by RT-qPCR and normalized to the levels of RLuc mRNA (Figure 6C). These experiments showed that downregulation of STAU1 or UPF1 increased the mRNA levels of FLuc-mWT and FLuc-M1 by ∼3.2- and 4.7-fold and 4.6- and 3.5-fold, respectively, compared with cells transfected with control siRNA (Figure 6D). Similar experiments were also performed using constructs into which each PBS core variant was inserted into the ΔPBS region of pFLuc-ΔPBS (Figure 6E). As expected, siRNA-mediated silencing of STAU1 or UPF1 increased the mRNA levels of FLuc-ΔPBS-mWT and FLuc-ΔPBS-M1 by ∼3.7- and 4.6-fold and 3.0- and 4.3-fold, respectively, compared with cells transfected with control siRNA (Figure 6F). It is particularly noteworthy that the insertion of other PBS core variants (M2, M3 and M4) downstream of FLuc or into the ΔPBS region of FLuc-ΔPBS did not significantly change the level of any of the corresponding mRNAs (Figure 6D and F). These data are also consistent with the STAU1 binding results shown in Figures 3 and 4 and support the idea that the SMD machinery recognizes the M2, M3 and M4 sequences much less efficiently than it does the mWT and M1 sequences. Thus, we established that the PBS core in the REQ 3′UTR is essential for SMD.

## DISCUSSION

In this study, we showed that the 3′UTR of REQ, along with its *trans*-acting factor STAU1, has the ability to induce REQ mRNA decay. We presented evidence that G8, a region of the REQ 3′UTR, is involved in the downregulation of REQ protein, concomitantly with the downregulation of REQ mRNA, by using a Tet-regulated transactivation system (Figure 1). By *in vitro* mutagenesis and RNA EMSAs with K562 cytoplasmic extracts (Figures 2 and 3), we identified the PBS core, which contains a putative stem–loop secondary structure, as a mediator for the binding of *trans*-acting factor(s) to the REQ 3′UTR. When the predicted stem–loop structure in the mouse PBS core was partially disrupted, the binding activity of these *trans*-acting factors was greatly reduced or eliminated (Figures 3 and 4 and Supplementary Figure S3). Next, we screened a K562 cDNA expression library with an RNA–ligand binding assay and identified STAU1 as a protein that binds to the REQ 3′UTR (Figure 4). And then, we established that REQ mRNA is regulated by SMD. The levels of both endogenous REQ mRNA and REQ protein were increased upon siRNA-mediated silencing of either UPF1 or STAU1 in mammalian cells, indicating that REQ mRNA is indeed a bona fide SMD target (Figure 5). Finally, we elucidated that the PBS core is necessary for SMD (Figure 6).

Recently, post-transcriptional regulation, especially when it is mediated by the mRNA 3′UTR, has been identified as a major mechanism of gene regulation that contributes to cell fate decisions during differentiation and development. At the same time, through studies that have employed RNA EMSAs, the functions of the 3′UTRs of many viral genomes have been elucidated (36–39). The majority of these studies have concluded that stem–loop structures in the 3′UTRs are important for translational regulation. The data presented in this study are also consistent with this pattern. We identified the PBS core, which contains a putative stem–loop secondary structure composed of an 8-bp central stem, a 1-bp mismatch in the middle, and a terminal stem–loop structure consisting of a 3-bp stem and a 4-nt loop, as a mediator for the binding of STAU1 to the REQ 3′UTR (Figures 3 and 4 and Supplementary Figure S3). Accordingly, the sequence identities shared by the PBS cores from various mammalian species are greater than 73% (Supplementary Figure S2C), and the putative RNA structures of the PBS cores of various mammals were all predicted to share a stem–loop harboring more than a 3-bp stem and a loop of varying sizes (Supplementary Figure S2D). It is also noteworthy that STAU1 does not seem to bind unstructured PBS (Figure 2D). Furthermore, we could demonstrate that STAU1 readily crosslinks to the structured RNA driven by the PBS of the REQ 3′UTR (Supplementary Figure S5B). Thereby, we concluded that STAU1 may bind the PBS core of the REQ 3′UTR by recognizing the RNA structure.

*Drosophila* STAU was reported to preferentially bind to stem structures in long 3′UTRs (40). Recent RNA deep sequencing analysis with crosslinking and RNA IP revealed that STAU1 binds to the highly defined and kinetically stable RNA secondary structures with stem–loop in a sequence-independent manner (41). Although the major STAU1 binding site is a few hundred nucleotides base pairs, a short stem–loop RNA structure is also a STAU1 binding site (41). Indeed, STAU1 preferentially binds to long base pairs up to ∼300 base pairs, but STAU1 is still capable of binding of the RNA structure that has more than one mismatch (35), suggesting that our predicted RNA secondary structure could be a potential binding site of STAU1. However, it is noteworthy that our STAU1 binding study on the PBS core is preliminary since we solely rely on RNA structure prediction software to define the structure of the element within the 3′UTR with a few of mutant constructs (Figure 3B and Supplementary Figures S2D and S3). Thus, further experiments will be required to conclusively establish the role of the putative RNA structure within the PBS core. It is also noteworthy that the binding of STAU1 to typical double-stranded RNA target sequences has been reported to be difficult to detect by UV cross-linking (41,42). However, we could easily detect not only cellular factors but also GST–STAU1–RNA complex by UV crosslinking using radiolabeled PBS in the REQ 3′UTR in reactions (Figure 2E and Supplementary Figure S5B). One possible explanation for this discrepancy is that although the PBS of the REQ 3′UTR requires double-stranded RNA helices for STAU1 binding, RNA–protein crosslinks may readily be formed between STAU1 and the adjacent single-stranded mismatch and/or loop sequences.

Several points should be emphasized from the perspective of REQ-mediated globin-switching. Although G8 was originally cloned as a putative globin-switching factor (10,11), the function of G8 in globin-switching is still controversial. We hypothesized that the overexpression of G8 may result in loss of cytoplasmic *trans*-acting factors that normally bind to the REQ 3′UTR, thereby increasing the level of REQ protein and directly or indirectly driving β-globin expression in γ-globin-expressing K562 cells. In addition, we have shown in the present study that REQ is subject to SMD (Figures 5 and 6). Since SMD has been shown to be an important mechanism regulating the expression of key genes involved in processes such as myogenesis (26), keratinocyte motility (35) and adipogenesis (24), it will be interesting to analyze whether SMD-mediated regulation of REQ expression is involved in globin-switching (or adult-type β-globin expression) in erythropoiesis. Furthermore, human REQ (DPF2) was recently identified as an efficient adaptor protein between the SWI/SNF complex and RelB/p52, thereby playing important roles in noncanonical NF-κB transcriptional activation and also in its associated oncogenic activity (8). REQ is also known to act as a nuclear receptor corepressor, selective for Estrogen-Related Receptor α (ERRα), by associating with both acetylated histone H3 and HDAC1 (43). Thus, it will also be interesting to examine whether REQ plays a transcriptional regulatory role as a component of the SWI/SNF chromatin remodeling complex or as a corepressor of globin gene expression in MEL cells.

## SUPPLEMENTARY DATA

Supplementary Data are available at NAR Online.

SUPPLEMENTARY DATA
