# Erratum

**DOI:** 10.1093/nar/gku668

**Published:** 2014-09-02

Staufen1-mediated mRNA decay induces Requiem mRNA decay through binding of Staufen1 to the Requiem 3′ UTR Min Young Kim, Jungyun Park, Jong Joo Lee, Dae Hyun Ha, Jonghwan Kim, Chan Gil Kim, Jungwook Hwang and Chul Geun Kim Nucl. Acids Res. (2014) 42 (11): 6999–7011. doi: 10.1093/nar/gku388.

First published online: May 5, 2014. Corrected after print: June 19, 2014.

The original version of this Article published on May 5, 2014, did not include the revised Figures 2 and 3 provided by the Authors after online publication. The Supplementary Figures had also been omitted. These errors have now been corrected in the new online version of the Article. The Publisher wishes to apologise to the Authors for these errors.

