# Supplementary material for: Staufen1-mediated mRNA decay induces Requiem mRNA decay through binding of Staufen1 to the Requiem 3′UTR
Source: Nucleic Acids Res. 2014 May 5;42(11):6999–7011. doi: 10.1093/nar/gku388 (PMC4066795; doi:10.1093/nar/gku388)
Supplement: SUPPLEMENTARY DATA [file supp_42_11_6999__index.html]

SUPPLEMENTARY DATA 

# Staufen1-mediated mRNA decay induces Requiem mRNA decay through binding of Staufen1 to the Requiem 3′UTR

## SUPPLEMENTARY DATA

**Files in this Data Supplement:**

- Supplementary Data
