# Supplementary material for: Staufen1-mediated mRNA decay induces Requiem mRNA decay through binding of Staufen1 to the Requiem 3′UTR
Source: Nucleic Acids Res. 2014 May 5;42(11):6999–7011. doi: 10.1093/nar/gku388 (PMC4066795; doi:10.1093/nar/gku388)
Supplement: SUPPLEMENTARY DATA [file supp_gku388_suppl_manuscript.docx]

**Article No.: gku388**

Manuscript No: **NAR-00194-Y-2014.R2**

**Staufen1-mediated mRNA decay induces Requiem mRNA decay through binding of Staufen1 to the Requiem 3’UTR**

Supplementary Material

Min Young Kim^1,$^, Jungyun Park^2,$^, Jong Joo Lee^1,$^, Dae Hyun Ha^1^, Jonghwan Kim^1, #^, Chan Gil Kim^3^, Jungwook Hwang^2,^*, Chul Geun Kim^1,^*

^1^Department of Life Science and Research Institute for Natural Sciences, College of Natural Sciences, ^2^Graduate School for Biomedical Science & Engineering, Hanyang University, Seoul 133-791, and

^3^Department of Biotechnology, Konkuk University, Chungju 380-701, Korea

Contents

Materials and methods

Supplementary references

Supplementary figure legends

**SUPPLEMENTARY MATERIALS AND METHODS**

**Plasmids**

To construct pTRE-EGFP, pEGFP-N1 (Clontech) was digested with NotI/SacII, and the NotI-digested site was subsequently blunted with Klenow DNA polymerase. The resultant NotI (blunted)/SacII fragment was then subcloned into pTRE (Clontech) cut with BamHI (similarly blunted) and SacII. Plasmid pTRE-EGFP-G8 was constructed by inserting the G8 region of mouse REQ cDNA, corresponding to bases +1,569 to +2,391 (i.e., from 431 bases downstream of the translation termination codon to the poly(A) sequence), in-frame with the 3’ terminus of EGFP and upstream of the pTRE-EGFP SV40 poly(A) signal sequence. Plasmids pGEM-G8 and pGEM-G8 (P1-P10) were constructed for the generation of *in vitro* RNA transcripts containing either the G8 region or a series of 5’ G8 deletion mutants. First, pGEM3-XhoI was constructed by inserting a XhoI site into pGEM3. Next, pGEM-G8 was constructed by subcloning the XhoI/SalI G8 cDNA fragment, obtained by digestion of pBK-CMV, into pGEM3-XhoI. To generate plasmids driving the expression of serial deletion mutants of G8, the Erase-A-Base system (Promega) was used according to the manufacturer's instructions. To generate exonuclease III-resistant 3' and 5' overhangs, the restriction enzymes SphI and SalI were used, respectively. To generate RNA probes P8-10, templates were initially prepared by PCR using the following combinations of primer sets: G8-688 (sense), 5’-CCT TAG TCC CTT TGC-3’; G8-830 (antisense), 5’-TGG CCC CCA AGG GC-3’; G8-802 (antisense), 5’-CGA GTT AAA AAA CAG GCA C-3’; and G8-754 (antisense), 5’-GGA AGA ACA AAA AGG G-3’. PCR products were directly subcloned into pT7Blue (Novagen) using the TA cloning method. To generate RNA probes for the wild-type human PBS, wild-type mouse PBS, and mutant versions of the mouse PBS (M1-M4), sense and antisense oligonucleotides containing the corresponding sequences were synthesized and used to generate double-stranded DNA molecules. These sequences were subcloned directly into pGEM-T (see Figures 2F and 3A for the corresponding sequences). Constructs driving the expression of the wild-type PBS core and mutant versions of the PBS core (M1-M4) were generated by subcloning the corresponding DNA fragments (liberated with XbaI and SpeI) from the appropriate pGEM-T-based parent constructs into XbaI-digested pGL3. To measure the effect of the PBS on mRNA decay, reporter constructs harboring the pGL3-promoter-REQ 3’UTR, -G8, or –poly(A) sequence were constructed by replacing the SV40 late poly(A) signal sequence with the corresponding PCR-amplified sequences, using the BamHI and XbaI sites. A forward primer introducing an XbaI linker and a reverse primer introducing a BamHI linker were employed in each PCR reaction. Primer sequences were as follows: re3u1f (5’- GAG CTC TAG Atg tgc cac cca gct ccc c-3’), re3u2f (5’-GAG CTC TAG Agg aag cag ctg gag tga g-3’), re3u3f (5’-GAG CTC TAG Aga cat taa gta gga ggg g-3’), and re3u4r (5’-CCC GGG ATC Cga ttt taa ccc aat ttt tgt a-3’). To construct pGL3-△PBS, two PCR products were generated using the G8N1-G8N2 and G8N3-G85R primer sets. The resultant products were subcloned into pGEM-T Easy, and the fragments liberated by SpeI/SacII digestion of the resultant vectors were subcloned in tandem into the promoter region of XbaI-digested pGL3. Primer sequences were as follows: G8N1 (sense), 5’-cac ttg cca gga cct ttt c-3’; G8N2 (antisense), 5’-AGC AAA GGG ACT AAG GAT GGG G-3’; G8N3 (sense), 5’-tgt tct tcc tga acc tgg gc-3’; and G85R (antisense), 5’-AAC TAA ATA TGC GAG GGG GGG CC-3’. Constructs driving the expression of mutant versions of the PBS core (M1-M4) were generated by subcloning the fragments liberated by SacII digestion of the appropriate pGEM-T-based parent vectors into SacII-digested pGL3-ΔPBS. The orientation of each insert was verified by either restriction enzyme digestion or colony PCR and subsequently confirmed by sequencing.

**Induction test of the Tet-on regulator**

After transient transfection of pTRE-Luc into individual K562 clones harboring p172-1, each clone was divided into duplicate cultures, and one culture of each clone was propagated in the presence of 1 μg/ml doxycycline. Transfected cells were harvested after 48 hr and lysed by the freeze-thaw method ([1](#_ENREF_1)). Insoluble material was pelleted by centrifugation at 1,000 rpm for 10 min at 4°C. Next, 50 μl (10 μg of protein) of supernatant was mixed with 50 μl of luciferin reagent, and the resultant light was measured using a luminometer (Lumat LB9510, Berthold). For further confirmation, cells were transiently transfected with pTRE-EGFP and treated with doxycycline as described above, and the EGFP-expressing cells were visualized using a fluorescence microscope equipped with a WIB filter (Olympus, BX-50).

**Flow cytometry**

Cells were harvested, washed twice with PBS, pelleted by centrifugation, and resuspended in 875 μl of PBS. Next, 125 μl of 2% para-formaldehyde was added, and cells were fixed for 1 hr at 4°C. After another two washes with PBS, fixed cells were analyzed on a Becton Dickinson FACScalibur platform using CellQuest^TM^ (Becton Dickinson) software.

***In vitro* transcription**

*In vitro* transcription was performed as described in the manufacturer's manual (Promega). For *in vitro* synthesis of RNA transcripts, deletion mutants of pGEM-G8 were linearized with XhoI, deletion mutants of pT7Blue-G8 were linearized with BamHI, and pGEM-T constructs encoding the wild-type PBS core or its mutants (M1-M4) were linearized with SpeI. Linearized vectors were subjected to *in vitro* transcription with either SP6 or T7 RNA polymerase (20 U; Böehringer Mannheim) in a 20 μl reaction containing 40 mM Tris-HCl (pH 7.9), 6 mM MgCl_2_, 10 mM NaCl, 2 mM spermidine, 10 mM dithiothreitol (DTT), 2.5 mM of each ribonucleotide (A, G, and U), 100 μM CTP, 200 μCi of [α-^32^P]-CTP (800 μCi/mmol; NEN), 100 μg of linearized DNA template, and 20 U of RNasin (Promega). Reaction mixtures were incubated for 30 min at 37°C. After the addition of 20 U RNase-free DNase I (Promega), reaction mixtures were incubated for another 15 min at 37°C. RNA probes were then precipitated and resuspended in 20 μl of distilled water. The size and integrity of each RNA probe was verified by electrophoresis on a 5% polyacrylamide gel, and the activity of each probe was measured with a scintillation counter (Beckman LS6500, USA).

Cell fractionation

Cellular extracts were prepared as previously described, with minor modifications ([2](#_ENREF_2),[3](#_ENREF_3)). Briefly, cells were chilled on ice for 5 min, washed twice with PBS, harvested by centrifugation at 150 x *g* for 5 min, and lysed in ice-cold lysis buffer containing 10 mM Tris (pH 7.4), 1.5 mM MgCl_2_, 10 mM KCl, 0.5 mM DTT, and 0.1 mM PMSF. Cells were lysed on ice by 20 pulses with a Dounce homogenizer. The efficiency of cell lysis was verified by visual inspection on a light microscope; nuclei were then removed by centrifugation at 5,000 rpm for 5 min at 4°C. Cell supernatants were removed and centrifuged at 100,000 x *g* for 1 hr at 4°C. The upper phase containing the soluble fraction was considered to be the cytoplasmic fraction.

**UV-induced crosslinking of RNA**

UV-induced crosslinking of RNA was carried out as previously described ([4](#_ENREF_4)), with minor modifications. Briefly, binding mixtures were treated with RNase for 15 min at room temperature, transferred to an ice bath, and irradiated with short-wavelength UV light (254 nm) at a distance of 3 cm for 10 min. The resultant UV crosslinked products were boiled in Laemmli sample buffer for 3 min and analyzed on a discontinuous 10% polyacrylamide-SDS gel. The gel was dried, and complexes were visualized by autoradiography. Prestained SDS-PAGE standards (Bio-Rad) were used to determine the relative molecular masses of the UV crosslinked products.

**Expression and purification of recombinant GST-hSTAU1**

Human STAU1 was recombinantly expressed as a GST-fusion protein in BL21(pLys) *Escherichia coli* cells and purified using Glutathione-Sepharose resin (Amersham-Pharmacia). Fusion protein integrity was confirmed by Coomassie blue staining and immunoblotting after SDS-polyacrylamide gel electrophoresis.

**RNA ligand-based cDNA expression library screening**

Phagemid-based K562 cDNA expression libraries were constructed by isolating mRNA from cells with an Ultraspec-RNA isolating system (Biotech) and a biotinylated oligo(dT) probe (Promega). Double-stranded cDNA was synthesized using a cDNA synthesis kit (Stratagene), and the resultant cDNA was ligated into the λZAP II express phage vector (Stratagene). G8 RNA-binding clones were identified using a screening assay for the detection of RNA-binding proteins as previously described ([5](#_ENREF_5)). Briefly, phagemids (1-1.5 x 10^4^ pfu/plate) were inoculated onto LB plates harboring a lawn of XL1-Blue cells (Stratagene). Following the binding of phage proteins to nitrocellulose membranes, the membranes were blocked in screening buffer (15 mM HEPES pH 7.9, 50 mM KCl, 0.1% [w/v] Ficoll 400-DL, 0.01% polyvinyl-pyrolidon PVP-40, 0.1 mM MnCl_2_, 0.1 mM ZnCl_2_, 0.1 mM EDTA, and 0.5 mM DTT) containing 0.1 mg/ml yeast RNA (Sigma, RNA type VI) in order to reduce nonspecific binding of the probe RNA. Specific RNA-protein interactions were detected by hybridization with the [α-^32^P]-labeled G8-RNA ligand. Nonspecific bound radioactivity was removed by washing the membranes four times for 5 min in 100 ml SB buffer. Membranes were then exposed to Kodak X-Omat AR film for 24 hr at -70^o^C. Putative positive clones were identified and purified to homogeneity in both secondary and tertiary screening rounds. Each positive phagemid was converted into a plasmid (pBK-CMV) by inoculating the phage into XLOR cells along with the ExAssist helper phage (Stratagene).

**REFERENCES**

1. Zambrano, N., Minopoli, G., de Candia, P. and Russo, T. (1998) The Fe65 adaptor protein interacts through its PID1 domain with the transcription factor CP2/LSF/LBP1. *The Journal of biological chemistry*, 273, 20128-20133.

2. Hel, Z., Skamene, E. and Radzioch, D. (1996) Two distinct regions in the 3' untranslated region of tumor necrosis factor alpha mRNA form complexes with macrophage proteins. *Molecular and cellular biology*, 16, 5579-5590.

3. Wang, X., Kiledjian, M., Weiss, I.M. and Liebhaber, S.A. (1995) Detection and characterization of a 3' untranslated region ribonucleoprotein complex associated with human alpha-globin mRNA stability. *Molecular and cellular biology*, 15, 1769-1777.

4. Hwang, Y.K. and Brinton, M.A. (1998) A 68-nucleotide sequence within the 3' noncoding region of simian hemorrhagic fever virus negative-strand RNA binds to four MA104 cell proteins. *Journal of virology*, 72, 4341-4351.

5. Sanger, F., Nicklen, S. and Coulson, A.R. (1977) DNA sequencing with chain-terminating inhibitors. *Proceedings of the National Academy of Sciences of the United States of America*, 74, 5463-5467.

**SUPPLEMENTARY FIGURE LEGENDS**

**Supplementary Figure 1.** The protein binding site in G8 interacts with one or more cellular factors. EMSAs were carried out using the cytoplasmic fraction of K562 cells and radiolabeled probes corresponding to various G8 deletion mutants. Assays were carried out either without **(A)** or with **(B)** RNase treatment (as in Figure 2A). Arrow heads, asterisks, and diamonds indicate RNA-protein complexes, unstructured RNA, and digested RNA fragments, respectively.

**Supplementary Figure 2.** The sequence and predicted secondary structure of the PBS core in the REQ 3’UTR is conserved among mammalian species. **(A)** Homology comparison of REQ 3'UTR sequences from mouse and other mammalian species. The appropriate cDNA sequences were retrieved from GenBank and aligned using CLUSTALW. **(B)** Homology comparison of the PBS sequences in the REQ 3’UTRs in mouse and other mammalian species. **(C)** Alignment of nucleotide sequences (left), and homology comparison (right) of the PBS core from the mouse REQ 3’UTR and those of other mammalian species. Dashes indicate gaps that maximize the alignment. Nucleotides identical to those in the mouse sequence are marked with a red shadow, whereas conserved sequences are denoted under the alignment with asterisks. **(D)** Computer-predicted RNA secondary structures (using the RNAfold web server) of the PBS cores from various mammalian species. The structure is colored by base-pairing probabilities. For unpaired regions the color denotes the probability of being unpaired.

**Supplementary Figure 3.** Computer-predicted RNA secondary structures (using the RNAfold web server) and the ΔGs of the corresponding mWT and mutant RNA sequences shown in Figure 3A. Central stem, mismatch and terminal stem-loop indicate the region in the predicted RNA secondary structure. The structure is colored by base-pairing probabilities. For unpaired regions the color denotes the probability of being unpaired.

**Supplementary Figure 4.** Detection of RNA binding-proteins using a radioisotope-labeled riboprobe. **(A)** Autoradiograph showing positive putative clones after primary screening. All RNA-binding proteins were additionally confirmed by secondary/tertiary screening. **(B)** Summary of clones identified by RNA-ligand screening of the K562 cDNA expression libraries using the REQ 3’UTR probe and their functions. **(C)** Western blot analysis confirming the expression of hSTAU1 in XL1-Blue cells transformed with pBK-CMV-hSTAU1. GST-hSTAU1 was used as a positive control; a crude cell extract of XL1-Blue cells was used as a negative control. The dot and the asterisk represent GST-hSTAU1 and hSTAU1, respectively.

**Supplementary Figure 5.** The protein binding site in G8 interacts with GST-STAU1. **(A)** EMSAs were carried out with recombinant GST-STAU1 and radiolabeled probes corresponding to various deletion mutants of G8. GST-STAU1 specifically recognized the PBS in G8. Arrow heads and asterisks indicate RNA-protein complexes and unstructured RNA, respectively. **(B)** GST-STAU1 was crosslinked with the radiolabelled PBS by UV. After UV crosslinking, samples were treated with RNase mixture followed by loading onto SDS-PAGE.

**Supplementary Figure 6.** siRNA-mediated silencing of UPF2 reduces the abundance of REQ mRNA. **(A)** Cell lysates were prepared from HeLa cells transiently transfected with either UPF2-specific siRNA or control siRNA. Cell lysates were analyzed by Western blotting with specific antibodies to determine the expression levels of UPF2 and REQ. Expression levels were normalized to the expression level of calnexin. Mean values with S.D. were calculated from three independent experiments. **(B)** Total RNA was prepared from HeLa cells transiently transfected with either UPF2-specific siRNA or control siRNA. RT-qPCR was then performed to determine the amount of REQ mRNA. REQ mRNA expression data are expressed relative to the mRNA level of the internal control gene, GAPDH.
